# Patient Engagement as Contributors in Online Health Communities: The Mediation of Peer Involvement and Moderation of Community Status

**DOI:** 10.3390/bs13020152

**Published:** 2023-02-10

**Authors:** Jun Wang, Tang Yao, Yani Wang

**Affiliations:** 1 School of Economics and Management, Beihang University, Beijing 100191, China; 2 Yanjing Medical College, Capital Medical University, Beijing 101300, China

**Keywords:** online health communities, patient engagement, social support, peer patient involvement, community status

## Abstract

This study focuses on patient engagement in online health communities (OHCs) and investigates the mechanism related to the impact of social support provided by patients on their personal engagement. Based on social support theory, we put forward a research model and conduct empirical analysis using datasets of 4797 patients with 160,484 posts and 1,647,569 replies from an online health community in China. The mediation of peer involvement and moderation of community status are also examined. The results indicate that the subdimensions of social support positively influence patient engagement with informational support exerting the greatest impact. Peer patient involvement imposes significant partial and positive mediating effects on the relationships, especially on informational support. Community status negatively moderates the impacts of social interactions and informational support on patient engagement in that the influence of social interactions and informational support are more profound for patients with low community status. The findings can bring an understanding of patient engagement in OCHs, and provide theoretical and practical implications to facilitate the development of an online healthcare service.

## 1. Introduction

With the advancement of Internet and Web 2.0 technology, online health communities (OHCs) have changed the way physicians and patients communicate about health-related knowledge. These communities facilitate participants establishing connections and sharing diagnosis experiences or emotional messages by supplying a large amount of tangible and intangible resources that are difficult to obtain in offline medical treatment [1]. Different types of OHCs for physician–physician, patient–patient or patient–physician interactions have been designated for various purposes and serve as important channels for participants to communicate with others [2,3,4]. In open OHCs, patients have the opportunity to share their experiences, feelings and therapy options in different ways, while others can respond by exchanging medical or clinical suggestions with their peers [5]. Community members’ participation is a vital indicator for the sustainability of OHCs and has obtained widespread concern among scholars. Patient engagement in OHCs has two aspects: seeking and sharing information, both of which are essential for successful healthcare outcomes [6]. Several studies have regarded patients as information seekers in virtual communities, but considered less about patients’ voluntary sharing information behavior, which is also highly associated with positive health outcomes [7,8]. Therefore, our research aims to focus on patients’ knowledge contribution behavior and uncover the mechanism of patient engagement in OHCs.

In this study, we identify the antecedents stimulating patient engagement in OHCs drawing upon social support theory. Social interactions represent initial steps affecting patients’ behaviors through relationship building because it can promote trust, cooperation and reciprocity among patients [9]. In patient–patient OHCs, patients can establish relationships and provide mutual assistance by sharing health-related experiences or suggestions. Informational support provides specific health-related information for patients to solve problems encountered in the process of diagnosis or treatment [10]. When a patient provides informational support in the form of concise records or advice, patients seeking solutions to enhance their health can obtain information directly. Emotional support in our research denotes a patient’s emotional tendencies in the support that they provide to others in the community [11]. In this regard, we first examine the linkage between the social support comprising social interactions, and informational and emotional support provided by patients and patients’ personal engagement in OHCs.

Meanwhile, the social support a patient provides may trigger other individual involvement. Other patients’ involvement can be considered to be both an outcome of the social support one patient provides and a powerful stimulus of his/her engagement in OHCs. Involvement reflects the perceived personal importance and relevance that consumers feel based on their needs and interests within the communities [12]. If a patient provides highly relevant social support, it will arouse other individuals’ concern and interest with the product or service. Furthermore, support seekers’ involvement can potentially accelerate the providers’ continuous engagement. Some scholars have confirmed the important connections between involvement and users’ decision making [13,14]. Based on the previous literature, we assume that peer involvement acts as a critical mediation variable in the relationship between social support and patient engagement. Consequently, the second research question explores when a patient provides social support in OHCs, whether and how the support seekers’ involvement can serve as a mediation variable in the above relationships.

In addition, we explore whether the community status of an information provider affects the strength of influential routes between social support and engagement. In communities, a provider with high status has the potential to attract more members to participate and further promote engagement. However, social support may signify the difference in contributing and accessing information among different population groups [14]. Therefore, community status can be considered as a moderating variable for the factors to exert effects. We operationally define patients’ community status as the mechanism represented by the points, money and fame a patient gains in the community. The third task is to examine whether and how the relationship between social support provided by patients and their subsequent engagement can be moderated by individual community status.

Our research deviates from earlier work in the following important ways. First, previous studies explored patient engagement regarding patients as information seekers, while we treat patients as information providers. Second, based on definitions of social support in the previous literature, we study social support from both structural and functional dimensions. Third, previous studies analyzed the influence of members’ involvement on their own engagement, while we investigate the mediating effect of other patients’ involvement on information providers’ engagement. Fourth, we treat patients’ community status as a moderator variable and examine its moderating effects on the relationships. The main findings can be summarized as follows. First, the two dimensions and three aspects of social support are positively related to patient engagement with informational support exerting the greatest impact and emotional support exerting the least impact. Second, involvement imposes significantly partial and positive mediating impacts on the relationships, especially on informational support. Third, the moderating effect of community status is only reflected in social interactions and informational support, and can attenuate their positive influence on patient engagement.

The rest of the paper is organized as follows. Section 2 introduces the current research concerning online health engagement, involvement and social support theory. A research model and corresponding hypotheses are developed in Section 3, followed by the research method including descriptions of data, variables and model specification. Empirical results and a discussion with the implications of our work are presented in Section 5 and Section 6, respectively. The last section displays the conclusion and limitations of this paper.

## 2. Literature Review

Online health communities (OHCs) have been widely recognized as platforms to promote communication among physicians and patients in the form of social support from either individuals or communities [14]. To understand patient engagement in OHCs, we employ social support theory combining online health engagement and patient involvement to study the influential paths from social support that patients provide to their own engagement.

### 2.1. Online Health Engagement

The concept of engagement has been widely discussed and given distinct definitions in various contexts. Online health engagement covers both the behavioral manifestation and psychological state of users. Engagement is described as behavioral manifestation when the community can grab members’ attention and they voluntarily join the group resulting from both internal and external motivation drivers [15]. Meanwhile, user engagement as a psychological state refers to individual willingness to contribute and assimilate when interacting with others in online communities [16,17]. Feng et al. [18] supported that engagement represents the extent to which users contribute to the communities to which they belong and proposed two dimensions of user engagement: participation in and recommendation of online communities to others. Ray et al. [19] defined engagement as the extent of dedication and an embedded personal value and capability that drives prosocial behavior. Based on the previous literature, we define patient engagement as patients’ behavioral manifestation and psychological state that unravels their willingness to invest time, attention and energy on the interactions resulting from internal and external stimuli during the interactive process, and can further drive optimistic healthcare outcomes in the specific online contexts.

Academics in online healthcare have studied the antecedents to forming engagement in OHCs. Feng et al. [18] verified the association between valanced social identities and engagement within OHCs by the perceptions of effectiveness using survey samples. Zhou et al. [6] explored the linkage between both intrinsic and extrinsic motivations and information providers’ voluntary behaviors based on motivation theory for mental health services. Gong et al. [20] summarized the factors influencing a patient’s physician selection by studying the influence of various information dimensions on patient trust from the physicians’ point of view. Lu and Zhang [8] found that information seeking behaviors can lead to patient compliance and encourage patients to actively seek health information and interact with each other in OHCs. Different to the previous research, we explore patient engagement in OHCs from the perspective of information providers.

### 2.2. Social Support Theory

Social support conveys providing social assistance to make the recipients feel they are cared for and loved, and are willing to perform the mutual obligations [21]. It could be facilitated by the transmission of information shared among individuals or groups. With the advancement of information technology, online interactions have become important sources of information sharing and people can share and acquire information to address the problems they have encountered or just have fun [22]. The major value and interest that users perceive from online community can be presented in forms of social support [23].

Social support is a multidimensional structure. According to Cobb [21], we define social support with structural and functional dimensions. Structural dimension denotes the nature and size of an individual social network, while functional dimension delineates the behavior interactions among supportive and supported members. Social interactions in OHCs contribute to members gaining assistance from their peers through frequent communications [24]. Mutual support among community members allows them to experience a sense of belonging and cohesion, leading to the willingness to enjoy their rights and fulfill their obligations in the community [22,25]. Virtual interactions often bring about two functional types of support: emotional and informational support. Emotional support describes an individual’s emotional tendency, including expressions of care or concern that an individual acquires from interactions with companions [26]. Information support indicates support provided in the form of information or suggestions, including assistance by providing intangible information and advice on solving problems [27]. Although information support is intangible, it provides valuable and necessary information assisting individuals to make accurate decisions and reduce uncertainty [28].

The frequent social interactions can enhance friendship and trust among patients, which may increase their intention to provide informational or emotional support, and further bring about subsequent engagement. In this study, we attempt to understand the effects of social support comprising social interactions, and emotional and informational support on patient engagement in online health contexts. We view social support as the resources provided by patients to assist others’ understanding of diagnosis and treatment information in OHCs.

### 2.3. Patient Involvement

Involvement refers to the degree of personal interest and relevance and is an important predictor of participation level in online social platforms [13]. According to Barki and Hartwick [29], involvement in information systems can be described as “a subjective state, reflecting the importance and personal relevance of a new system”. Involvement in OHCs reflects the extent to which people recognize that the community is personally important and relevant so that they are willing to participate in the shared health decision making [30]. It is the premise of engagement and is closely associated with the personal degree of participation during the interactions [12]. In OHCs, a high level of patient involvement can contribute to benefits such as more confidence in patient safety [31], mutual trust between patients and physicians [32] and an overall improvement in health status [33]. Moreover, patients with higher involvement can actively interact with others in the health decision process and show a strong willingness to engage in the groups [34]. Wang et al. [35] found that individuals search for various types of social support when they are at different levels of involvement, which can influence patient engagement in OHCs. Van et al. [36] confirmed that patient involvement exerts an important role on the interactions and value co-creation among physicians and patients in OHCs.

Patient involvement in this study denotes the psychological identification process of patients in the social interactions; that is, other patients’ feedback or reactions when a patient initiates a conversation. We assume that the connections between the social support provided by patients and their engagement may take effect, combining the involvement of other patients in the community. Consequently, we explore the mediating effect of peer involvement in the influential effects.

## 3. Research Framework

**Figure 1:**
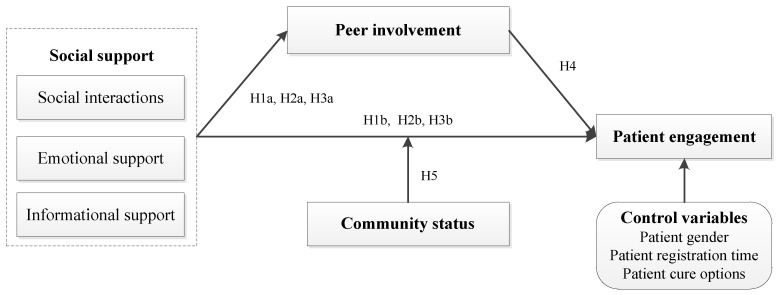
Research framework.

This study explores how social support provided by patients comprising social interactions, and emotional and informational support can promote patient personal engagement in OHCs, building on social support theory. The mediation of peer involvement and the moderation of community status are also investigated in the model. Moreover, we include patient gender, registration time and cure options as control variables because they may exert effects on patients’ personal engagement to some extent. The research framework is displayed in Figure 1 and hypotheses are discussed in the following sections.

### 3.1. The Effects of Social Support

In OHCs, social support usually means mutual support by means of social communications to make community members feel that they are being cared about and esteemed, and thus are willing to perform obligations when interacting with others in the group [37]. As one of the key aspects of social support, social interactions represent that a person builds relationships with others and generates perceptions of belongingness and dependence within the community, which will contribute to positive health outcomes [11]. Social interactions imply the desire of being recognized or helped by others within the social groups [35]. Therefore, patients are willing to trust the platform and exchange their suggestions in the group. Frequent social interactions can lead to positive attitudes of patients towards the communities, promoting their identification and assimilation with the community [38]. Moreover, patients will be infected and generate strong desires to retain long-term good relationships with the community. In this paper, we argue that social interactions established by patients positively influence peer patients’ perceptions of the importance and relevance of health communities, further promoting their own engagement in the communities. Therefore, we assume that:

**H1a:**  *Social interactions established by patients in OHCs are positively related to peer patient involvement.*

**H1b:**  *Social interactions established by patients in OHCs are positively related to their own engagement.*

OHCs provide patients and physicians with high levels of accessibility and social connectivity so that they can express their views about a disease or diagnosis options with each other anytime and anywhere [39]. Emotional support denotes the provision of concern, sharing happiness or sadness, and can occur alone or in conjunction with informational support by expressing care and understanding [40]. In OHCs, patients with health needs usually expect to obtain emotional support such as encouragement and empathy, and a sympathetic response from others with similar symptoms [41]. They can open their minds to people understanding them and providing concern, which can reduce their feelings of isolation and loneliness. If other patients feel this resonance, they may treat themselves as members of the community and provide support for reciprocal purposes, which will increase their sense of belonging in the community [42]. In our study, emotional support refers to the proportion of positive emotions in patients’ support during the interactions, which can help others to find a sympathetic response within the community. We assume that the emotional support provided by patients positively affects other patients’ personal perceptions of relevance and importance within the group, further promoting patient engagement within an online health community. Thus, we assume that:

**H2a:**  *Emotional support provided by patients in OHCs is positively related to peer patient involvement.*

**H2b:**  *Emotional support provided by patients in OHCs is positively related to their own engagement.*

Informational support in OHCs denotes related knowledge such as suggestions, guidance and useful information that community members provide to help others solve their problems or make good decisions [37]. In the communities, patients can present their symptoms, treatment experience and even questions that may be valuable information to others. When patients encounter similar health problems, they tend to ask for help from the platforms and search for explanations or answers they need in OHCs. Patients receiving the information will be more likely to have confidence in the treatment and form a further feeling of trust towards the information providers [43]. In general, informational support enables patients to obtain assistance from others’ messages and suggestions when solving their own health problems [22]. Such benefits increase patients’ involvement and subsequent engagement [35,36]. In the repetitious and interactive process of exchanging information, members’ involvement will be enhanced [12]. In this paper, we consider that informational support increases the involvement of other patients, which further promotes the engagement of the patients themselves. Consequently, we propose the following:

**H3a:**  *Informational support provided by patients in OHCs is positively related to peer patient involvement.*

**H3b:**  *Informational support provided by patients in OHCs is positively related to their own engagement.*

### 3.2. Patient Involvement as the Mediator

According to Barki and Hartwick [29], patient involvement in OHCs denotes patients’ belief about their personal relevance and importance of the community. From the behavioral manifestation, it can predict the degree of user engagement in OHCs with higher levels of involvement contributing to high engagement [12]. At the same time, online patients are different from those offline because they can interact at any time and any place with others [4,5]. Patients with different extents of involvement will seek various amounts of social support, which further influences their own engagement [35]. Previous research has found that members with high levels of involvement are more likely to contribute their own experiences and time to the community than others [6,30]. Patients’ frequent community visits and interactions can increase the provision of social support, leading to higher levels of involvement of other patients. Furthermore, other participants’ involvement is more likely to stimulate the initial patients to integrate into the group [37,38]. Specifically, other patients’ involvement enables providers of social support to psychologically recognize the community and contribute their information and emotions to the healthcare activities because of the participation of peer patients [44]. Consequently, we particularly investigate the mediating role of other patients’ involvement on information providers’ engagement and hypothesize that:

**H4a:**  *Peer patient involvement is positively related to the support providers’ engagement in the OHCs.*

**H4b:**  *The impacts of the social support that patients provide on their own engagement are mediated by peer patient involvement in OHCs.*

### 3.3. Community Status as the Moderator

Patients in online medical services are not only recipients of medical services but also providers and disseminators of medical resources. Information needs and contributions are the primary purpose of patient engagement in the community. Sufficient information searching can reduce patients’ health uncertainty and ensure easy access to information, which will exert a critical impact on healthcare outcomes [45]. Studies have demonstrated the importance of community status in knowledge contribution. Yang and Ju [46] found that physicians’ offline status (professional titles in a hospital) positively influences their online contribution behaviors. The monetary rewards a physician gains can also extrinsically motivate physician engagement in OHCs [4]. Liu et al. [47] verified that disparity in professional seniority can discourage physicians from contributing knowledge in OHCs. Feng et al. [18] indicated that users’ positive social identity can exert positive influences on their participation and recommendation intentions by the survey data from 221 users. In this study, we suppose that the influence of social support may vary according to the support providers’ community status. The money, points or fame one patient owns in the community are important signals urging individuals to participate, meaning that patients will be stimulated by the factors to engage in OHCs [6,48]. We expect community status to exert a moderating effect on the above relationships and hypothesize that:

**H5:**  *The impacts of the social support that patients provide on their own engagement are moderated by individual community status in OHCs.*

## 4. Research Method

### 4.1. Data Collection

Empirical research datasets were collected from Sweet Home, a well-known diabetes-related online health community in China. Sweet Home is an interactive platform that focuses on patient–patient interactions integrating online questions and answers, emotional communications and social sharing for diabetics with peer patients. Patients in the community can exchange their opinions and suggestions, express their affective tendencies or share their opinions and views of diagnosis and treatment, which enables subsequent patients to be involved as they are informed. Consequently, Sweet Home offers an appropriate context for us to investigate patient engagement in OHCs. We selected patients based on several popular forums including “Type 2 Diabetes”, “Diabetes Consultation” and “Type 1 Diabetes” because members in these sections are active and the total number of posts exceeds 500,000 every day. Therefore, datasets from these popular posts served as representatives of patient engagement.

In the data crawling stage, we first obtained all the related patients who participate in the community and recorded the basic information of these users in the form of dictionary, including patient name and patient id, and stored all the dictionaries in a large list for subsequent information crawling:

{‘name’:XXX,‘uid’:XXX}

**Figure 2:**
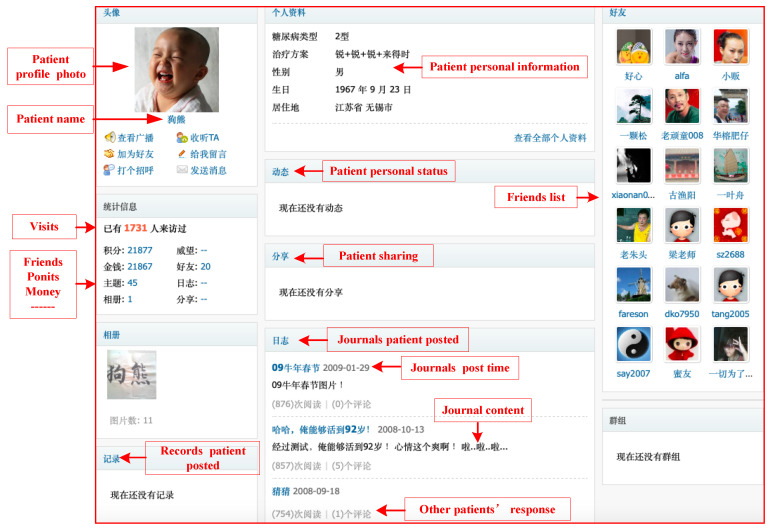
An example of the patient’s homepage.

**Figure 3:**
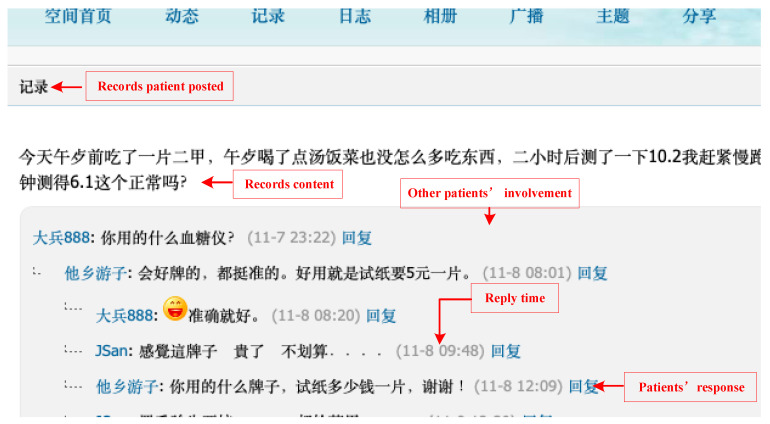
An example of other patient’s involvement.

We ensured most patients had frequent participation experience within the community in the past two years so that the personal and text information obtained was timely and effective. This method avoids the situation of randomly identifying or many “zombie accounts” when acquiring all patients in the community. The final filtered datasets came from patients who had good records of participation; that is, people who had posted and responded to others in community activities. After determining the patient range, we searched personal information and text information of all past postings and replies based on the patient’s homepage. A total of 4797 users with 160,484 posts and 1,647,569 replies from June to September in 2020 were identified. We further conducted data cleaning and removal of samples containing too much null value, datasets of 3709 patients were finally included in the analysis. Figure 2 shows the patient’s homepage in the community and Figure 3 shows the responses of other patients when a patient posts a record.

### 4.2. Variables and Measurements

#### 4.2.1. Dependent and Independent Variables

We conceptualized variables in the model and operationalized them using the crawled datasets. The dependent variable of patient engagement is measured by the volume, frequency and quality of patient replies. The volume of patient replies can be calculated by counting patient’s reply information, and the frequency and quality of replies are calculated by reply time and reply text, respectively.

The frequency of patient replies is reflected by the median time between patient response behaviors, namely:

$$\bar{t}=median\left\{t_{1,\;}\;t_{2,\;}\cdots ,\;t_{n}\right\}$$

The quality of patient replies is identified and scored by establishing a word database related to diabetes. We obtained professional vocabulary that contains 31,044 related vocabulary from the existing diabetes-related academic papers and topic discussion sentences. The number of words related to diabetes in user’s sentence is marked as the weight of each sentence through word segmentation, namely:

$$w_{i}=\sum k_{i},k_{i}=\left\{\begin{array}{c} 1,\;k_{i}\;is\;in\;P\\ \;0,k_{i}\;is\;not\;in\;P \end{array}\right\},\mathrm{where}\;P\;\mathrm{is}\;\mathrm{word}\;\mathrm{dictionary},{\;k}_{i}\;\mathrm{is}\;\mathrm{word}.$$

Consequently, the average value of the weights in patient’s personal reply sentences is used as the quality value, namely:

$$\bar{w}=\frac{1}{n}\sum_{i=1}^{n}w_{i},\;{\mathrm{where}\;w}_{i}\;\mathrm{is}\;\mathrm{the}\;\mathrm{weight}\;\mathrm{of}\;\mathrm{the}\;\mathrm{ith}\;\mathrm{sentence}.$$

In the independent variables, we considered the volume of patient’s friends and visits to be more suitable for representing patients’ social interactions with others. Emotional support reflects the proportion of positive emotions in the information support provided by patients and is measured using the emotion tendency contained in patient’s contents. Since patients can share their own life, diet, exercise methods and emotional content in the community, whose language style is similar to those in Weibo, we selected the emotion-related dataset from Weibo. We employed 370,000 sentences with positive and negative emotions as the training set and conducted logistic regression to train the model. We performed word segmentation on the text contents and put them into models to obtain the emotion category of each sentence. Finally, emotional support of each patient is calculated by the ratio of positive emotional statements to the total statements.

$$emotion=\frac{ep}{ep+en}$$

where *ep* is the volume of positive emotional statements and *en* is the volume of negative emotional statements of the total text contents.

#### 4.2.2. Mediating and Moderating Variables

In terms of the mediating variable, we argue that when a patient posts a review in the community, other patients’ responses will motivate him/her to keep following. That is, the effective involvement of other patients will have an impact on information providers’ engagement. In our study, patient involvement behavior is comprehensively calculated by the mean value of reply emotion tendency, time and quality of peer patients.

In order to clearly distinguish the impacts of social support from different patients, we specifically considered community status of patients as the moderator to take effect. Studies have confirmed the moderating role of social status in online context [49]. Compared with low status, members with high status will promote more participation. Patients’ community status in our study was operationalized into high and low to test whether different levels of patients produced different engagement. Moreover, patient’s gender, registration time and cure options were regarded as control variables for the analysis. All variables were standardized before analysis to make different variables actionable. Table 1 delineates the variables and relevant measurements utilized in this research.

### 4.3. Model Specification

The explanatory variable X includes the subdimensions of social support: social interactions, and emotional and informational support. The mediating variable M is peer patient involvement, the moderating variable W is patient’s community status and the dependent variable is patient engagement. The following models are established according to Baron and Kenny [51]:

(1) $$Patient\;engagement=c_{0}+\beta_{1}*Control\;variables+c_{1}*Social\;support+\varepsilon$$

(2) $$Peer\;involvement=a_{0}+\beta_{2}*Control\;variables+a_{1}*Social\;support+\varepsilon$$

(3) $$\begin{array}{l} Patient\;engagement\\ \;=c_{0}^{\prime }+\beta_{3}*Control\;variables+c_{1}^{\prime }*Social\;support+b_{1}*Peer\;involvement+\varepsilon \end{array}$$

(4) $$\begin{array}{l} Patient\;engagement\\ \;=c_{0}^{\prime }+\beta_{4}*Control\;variables+c_{2}^{\prime }*Social\;support+d_{1}*Social\;support\\ \;*\;Community\;status+\varepsilon \end{array}$$

Equation (1) refers to the main effects of social support provided by patients on patient personal engagement; Equation (2) denotes the impacts of social support on peer involvement; Equation (3) represents the mediating effect of peer involvement; and Equation (4) displays the moderating effect of community status. In the equations, $c_{1}$, $a_{1}$,$\;c_{1}^{\prime }$, $c_{2}^{\prime }$, $b_{1}$ and $d_{1}$ represent the parameters to be estimated.

## 5. Results

### 5.1. Descriptive Statistics

Before the empirical analysis, we first calculated the variables in the model and performed descriptive statistical analysis for the datasets. Table 2 provides the mean and standard deviation of the overall standardized variables and correlation matrix of all variables. The results show that the correlation coefficients of variables are all between 0 and 0.500. In addition, we performed VIF estimation and the maximum value is 1.45, as suggested by [52], so there is no serious multicollinearity problem in the model.

### 5.2. Empirical Analysis

#### 5.2.1. Results of Main Effects

We first conducted ordinary least squares (OLS) regressions to test the hypothesis concerning the main effects. Table 3 reports the results including the estimated unstandardized coefficients and some fit metrics for the models. Hypotheses 1, 2 and 3 examine the influence of social support that patients provide on peer involvement and patient personal engagement. The impacts of social support on peer involvement are positive and significant with the positive coefficients of 0.066 (*p* < 0.05), 0.010 (*p* < 0.05) and 0.182 (*p* < 0.001), respectively, after controlling for the effects of patient gender, registration time and cure options, as shown in Model (2). The findings also demonstrate that social support exerts a significantly positive influence on patient engagement with the regression coefficients of 0.160 (*p* < 0.001), 0.005 (*p* < 0.001) and 0.229 (*p* < 0.001) in Model (4). The results support all the hypotheses. A larger proportion of the variance of 25.8% in social support provided by patients contributes to their personal engagement. Hypothesis 4a examines the effects of peer involvement on patient engagement. The results indicate that peer involvement positively influences patient engagement (β = 0.008, *p* < 0.1), as shown in Model (5). Therefore, H4a is supported. When the mediator of peer involvement is taken into account, social support still exerts significantly positive effects on patient engagement, indicating that peer involvement partially mediates the linkage between social support and patient engagement.

#### 5.2.2. Results of Mediating Effects

Furthermore, we performed mediation analysis using a bootstrapping technique (*n* = 5000 bootstrap resamples, Model 4) based on an empirical estimation of the sampling distribution concerning the indirect effect [53]. The results of the indirect effects in Table 4 reveal that social interactions exert a significantly positive and indirect influence on patient engagement (effect = 0.003, LLCI: 0.001, ULCI: 0.006). In addition, the indirect effects of emotional and informational support are 0.000 (LLCI: 0.000, ULCI: 0.001) and 0.005 (LLCI: 0.001, ULCI: 0.008), respectively. Peer involvement imposes the greatest mediating effect on the relationship of informational support, while it has the least mediating effect on emotional support, indicating that informational support is more likely to promote patient engagement through peer involvement. The results of the mediation analysis show that when patients provide social support, the involvement of other patients or physicians can promote patients’ subsequent engagement.

#### 5.2.3. Results of Moderating Effects

We also conducted OLS regressions including the main effects model and moderated regression approach to investigate the moderating effect of community status, as shown in Table 5. The coefficient for the interaction of social interactions and community status is significantly negative (β = −0.916, *p* < 0.001) and the interaction of informational support and community status is −0.629 (*p* < 0.001), indicating that community status negatively moderates the effects of social interactions and informational support. The effects of social interactions and informational support on patients’ subsequent engagement are higher among patients with low community status than patients with high community status. One possible reason may be that low-status patients care more about interactions with others. They not only provide social support but also pay attention to its effects and evaluations by other patients, whereas patients with a high status care less about what others think about their support. However, the coefficient for the interaction of emotional support and community status is statistically nonsignificant (β = −0.020, *p* > 0.05), indicating no difference among patients with either high or low community status for the effect of emotional support. Therefore, H5 is partially and conversely supported.

**Figure 4:**
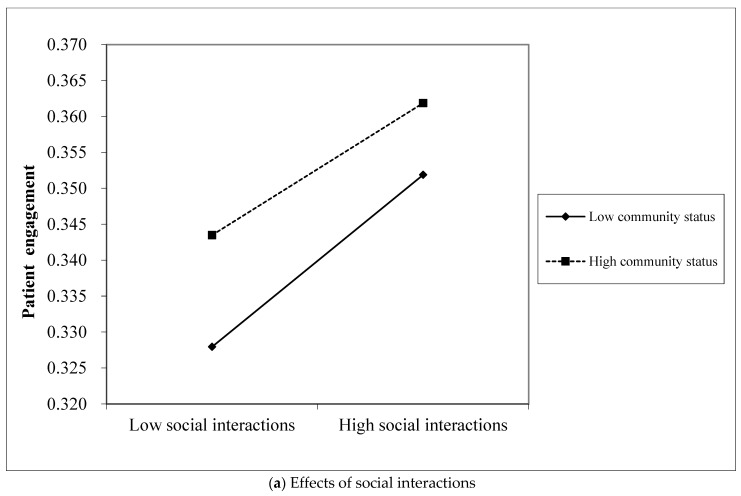

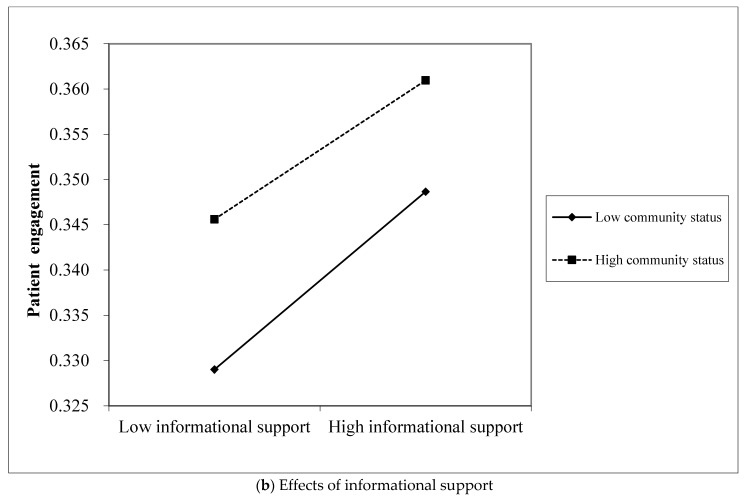
Moderating effects of community status.

We further performed graphical analysis to interpret the moderation effect of community status, as depicted in Figure 4. Figure 4a demonstrates the effects of social interactions at both high and low levels of community status (Mean ±1 SD). When patients have a low community status, the positive relationship between social interactions and patient engagement is significantly boosted (effect = 0.359, *p* < 0.001), while, when patients have a high community status, the effect of social interactions on patient engagement is relatively weaker (effect = 0.276, *p* < 0.001). Figure 4b illustrates the influence of informational support on patient engagement at the two levels of community status. The results reveal that when patients have a low community status, the positive relationship between informational support and patient engagement is significantly boosted (effect = 0.410, *p* < 0.001); when patients have a low community status, the effect of informational support on patient engagement is relatively weaker (effect = 0.320, *p* < 0.001). We can conclude that a high community status can bind the positive effects of social interactions and informational support on patient engagement.

### 5.3. Robustness Analysis

We tested the robustness of the main effects by employing the following approaches. First, considering that the dependent variable of patient engagement is calculated by initial count variables, we ran the models using negative binomial regression. The results are displayed in Table 6 and are mostly consistent with the results of the OLS model. Second, since the volume of patient replies may intuitively represent patient engagement, we substituted it for patient engagement as the dependent variable in the models. The results are shown in Table 7 and are consistent with the results of the OLS model, but the explanatory power of the models is not as good as that of the OLS models. Third, to better measure patient engagement, we reselected the samples and analyzed only those patients’ samples whose registration time was above the mean value. We found that the results are consistent, indicating that there is no sample bias, as shown in Table 8.

## 6. Discussion

With the prevalence of OHCs, patients have the opportunity to provide health-related information online in addition to that of physicians. This study is valuable for understanding the mechanism of how the social support provided by patients shapes their engagement in OHCs. Drawing upon social support theory, we conduct a research model and put forward hypotheses to depict the mechanism of patient engagement. The mediation of peer involvement and moderation of community status are also considered. Datasets from Sweet Home are utilized to verify the model and hypotheses. Results can be concluded as follows:

First, the social support provided by patients can play an important role in their engagement. The results demonstrate that patient engagement is highly related to informational support when all the factors are taken into account, indicating that the informational support that patients provide plays an important and fundamental role in patient engagement. Informational support in an online context is often presented in forms of information available to members, such as suggestions and advice to help others enjoy a good experience or make good decisions [24]. Consequently, both physicians and patients should place a greater emphasis on informational support. Meanwhile, social interactions among patients are also critical to patient engagement. Previous studies have investigated the effectiveness of social networks and social interactions on satisfying patients’ needs, suggesting that it is critical to stimulate interactions among patients by providing them with opportunities to participate in activities [47]. When patients are in a caring, trustworthy and respectful online environment, interpersonal relationships will be facilitated and informational exchange will frequently occur [54].

Second, peer involvement acts as an important and partial mediating mechanism in the relationships. Specifically, peer involvement exerts a greater mediating effect for informational support than for social interactions and emotional support. Informational support that patients provide can be more likely to influence patient engagement through peer involvement. Many studies in the IS literature have confirmed the significant mediating role of involvement in members’ participation in online communities [35,36,44]. When patients initiate a discussion, the response of peers can give them a sense of accomplishment, which can stimulate their desire and responsibility to contribute knowledge and engagement. Consequently, researchers should pay more attention to patient involvement as being complementary to healthcare social support.

Third, our study also sheds light on the interesting moderating effects of community status in the relationships. Specifically, community status can attenuate the positive effects of social interactions and informational support, indicating that low-status individuals may put in more effort to increase their levels of engagement by implementing more interactions with others. Patients with a lower community status could increase their participation by interacting with others and providing informational support, while high-status patients do not need to promote their engagement in this way. Consequently, community status may act as a useful part that can condition the influence of social support. In addition, we find that the moderating effect of community status on emotional support is nonsignificant, meaning that the moderation of community status is only reflected on social interactions and informational support but not on emotional support. For both low and high status, the influence of emotional support on patient engagement is almost unanimous.

### 6.1. Theoretical Implications

Our study makes several important theoretical implications. First, we enrich the current online health literature by proposing a fundamental but unexplored issue in terms of patient engagement from the perspective of patient–patient interactions. OHCs greatly shift the diagnosis environment for information sharing and interactions for both physicians and patients [4]. The current research in the IS domain has mainly focused on physician–physician or patient–physician interactions [2,6], but has considered patient–patient interactions less. We extend the extent literature on user engagement in OHCs to the point of patient–patient interactions and address the major question concerning how to stimulate patients’ sustainable engagement when they provide support in the community. Moreover, different from a few studies that explore patient engagement considering patients as information seekers in the community [8,55], we study patient engagement regarding patients as the information providers. The novelty of this research perspective could help scholars and managers develop a good knowledge of patient engagement in OHCs.

Second, this study clarifies the influential mechanisms through which social support provided by patients can influence their personal engagement in an online health context. Building on the online healthcare literature and social support theory, we develop a comprehensive research model in which social support comprising social interactions, and emotional and informational support are the antecedents of patient engagement, which could lead to better knowledge of the critical role of social support on shaping patient engagement. We prove that each of the three aspects of social support provided by patients can promote their own engagement, among which informational support and social interactions play fundamental and important roles on ensuring the sustainable development of communities. The model proposed in this study covers socially relevant factors of patient engagement, which will realize perfect integrations with current patient engagement and e-health research to give inspiration for future research [50].

Third, this study confirms the mediation of peer involvement and the moderation of patients’ community status on the influencing relationships. Information seekers’ involvement significantly and partially mediates the relationships, with informational support more likely to influence patient engagement through peer involvement. Community status is addressed in our research because patients may produce different behavioral motivations when they are under different statuses. The findings demonstrate that individuals with low status need to put more effort in to increase their engagement by implementing more interactions and providing more informational support for others. The findings can be employed to encourage information providers and seekers to enhance communications and interactions to augment mutual engagement, which will affect their subsequent health-related activities.

### 6.2. Practical Implications

Several managerial implications for OHCs also emerge from the findings. First, the results uncover the importance of patients’ knowledge contribution and support in fostering patients’ own engagement in OHCs. Individuals possess an inherent need to interact with one another whether online or offline [13]. As such, social interactions are fundamental and at the core of patient engagement. Through social interactions with others, patients can perceive a high sense of dependence and belongingness in the community [37]. Consequently, online health practitioners can implement appropriate strategies to stimulate patient interactions if they want to increase patient adherence and the value of community [22]. For example, community managers can design dedicated forums and small videos based on the latest hotspots to encourage members to communicate and interact. The activities contribute not only to enriching patients’ physical well-being but also to increasing patients’ cohesion with the communities.

In addition, practitioners should recognize the importance of peer patient involvement in empowering the co-creation of communities. This study indicates that informational support provided by patients is more likely to promote their engagement when other patients are involved in the interactions. If a patient provides informational support in the community and other patients give timely feedback, these behaviors will in turn arouse the patient’s desire to engage. Consequently, community managers should strive to increase the relevance of individuals and make patients feel their own relevance and importance in the community to ensure they have a sense of belonging and ownership [26]. Communities can design algorithms comprehensively considering individual characteristics and online behaviors, and develop recommendation systems to help patients conveniently access others with similar symptoms [27]. Discussion groups can also be created to improve peer patient involvement.

Finally, the results indicate that managers should focus on patients with high status and inspire them to provide support for the community. The results demonstrate that a high community status can attenuate the positive effects of social interactions and informational support. A high community status endows patients with a higher reputation and they may have other resources or advantages to demonstrate their engagement than members of low community status in OHCs. This is a reminder for managers to help them understand the mechanism and try their best to create a more complete mechanism to mobilize high status patients’ engagement. For example, they can take advantage of community incentives to stimulate patients’ eagerness, which may bring about unexpected outcomes.

## 7. Conclusions and Limitations

This study aims to explore how the social support provided by patients influences their own engagement in OHCs. We propose a research framework and hypotheses to explore the underlying mechanism concerning how the social support that patients provide influences their personal engagement. Datasets from Sweet Home are used to verify the model and hypotheses. The findings demonstrate that the three subdimensions of social support exert significantly positive effects on patient engagement, with informational support exerting the greatest influence and emotional support exerting the least impact. Specifically, peer involvement presents partially mediating impacts on the relationships with the greatest impact on informational support. A high community status can attenuate the positive effects of social interactions and informational support. The study can extend the current literature by providing a novel insight and offering implications for both scholars and managers to gain knowledge of how the social support that patients provide promotes patient engagement in OHCs.

This study also has several limitations. First, our research background is based on Sweet Home, a famous online health community in China. However, it will also be interesting to examine if patients in other platforms from different cultures experience the same effects. Second, although the model exhibits strong explanatory power and good prediction performance of social support on patient engagement in OHCs, the text analysis can still be improved into a more accurate analysis method that includes more factors including text quality and text emotion to obtain more universal research results. Third, when calculating model variables with different explicit variables, we do not consider the weight of different explicit variables and treat them equally. Scholars can take the weights of different factors into consideration in future research.

## Figures and Tables

**Table 1:** Description of variables.

Variable Type	Variables	Measurements
Dependent variable	Patient engagement [5]	We use the volume and quality of patient replies, and the median time between patient replies to measure patient’s engagement behavior.
Independent variables	Social interactions [21,26]	We use the volume of visits to the patient’s homepage and patient’s friends to measure patient’s social interactions.
	Emotional support [40,41]	We use the ratio of positive emotional statements to total statements that patients provide to measure patient’s emotional support.
	Informational support [22,39]	We use the volume of records, journals and threads posted by the patient to measure patient’s informational support.
Mediator	Peer involvement [44,50]	We use the percentage of positive sentiment, the average reply time and the quality of reply by peer patients to measure peer patient’s involvement.
Moderator	Community status [18]	We use the volume of points, fame and money patients obtained in the community to measure patient’s community status.
Control variables	Patient gender	Gender is coded with “0” for male and “1” for female.
	Registration time	Registration time refers to the amount of time since a patient registered for the community account to the date when we collect data.
	Cure options	Whether the patient discloses individual personal cure options.

**Table 2:** Descriptive statistics and intercorrelations.

	Variables	Mean	*SD*	1	2	3	4	5	6
1	Social interactions	0.010	0.033	1.000					
2	Emotional support	0.361	0.389	0.198 **	1.000				
3	Informational support	0.007	0.024	0.458 **	0.143 **	1.000			
4	Peer involvement	0.522	0.060	0.097 **	0.107 **	0.111 **	1.000		
5	Community status	0.008	0.028	0.467 **	0.155 **	0.436 **	0.115 **	1.000	
6	Patient engagement	0.346	0.021	0.413 **	0.246 **	0.410 **	0.095 **	0.324 **	1.000

Note: ** *p* < 0.01.

**Table 3:** Results of the main effects.

Dependent Variables	Peer Involvement		Patient Engagement		
	Model (1)	Model (2)	Model (3)	Model (4)	Model (5)
Constant	0.513 ***	0.512 ***	0.340 ***	0.339 ***	0.335 ***
Patient gender	−0.001	−0.001	0.001	0.000	0.000
Registration time	0.018 **	0.013 *	0.009 *	0.000	0.000
Cure options	0.010 **	0.003	0.008 *	0.003 *	0.003
Social interactions		0.066 **		0.160 ***	0.160 ***
Emotional support		0.010 **		0.005 ***	0.005 ***
Informational support		0.182 ***		0.230 ***	0.229 ***
Peer involvement					0.008 *
df	3	6	3	6	6
F	18.340	15.889	87.958	214.528	184.315
R^2^	0.015	0.025	0.066	0.258	0.258
Adjusted R^2^	0.014	0.024	0.066	0.257	0.257
$\Delta R^{2}$		1%	5.2%	23.3%	23.3%
Sample size	3709	3709	3709	3709	3709

Note: * *p* < 0.1, ** *p* < 0.05, *** *p* < 0.001. Unstandardized regression coefficients are reported.

**Table 4:** The mediating effects of peer involvement.

Indirect Path	Effect	Boot SE	LLCI	ULCI
Social interactions → Peer involvement → Patient engagement	0.003	0.001	0.001	0.006
Emotional support → Peer involvement → Patient engagement	0.000	0.000	0.000	0.0001
Informational support → Peer involvement → Patient engagement	0.005	0.002	0.001	0.008

Note: Bootstrap samples = 5000, Boot SE = bootstrap standard error, level of confidence = 95%, LLCI = lower limit of confidence interval, ULCI = upper limit of confidence interval.

**Table 5:** The moderating effects of community status.

Dependent Variables	Patient Engagement		
	Baseline Model	Main Effects	Moderation Effects
Constant	0.340 ***	0.339 ***	0.339 ***
Patient gender	0.001	0.339 ***	0.000
Registration time	0.009 *	0.000	−0.001
Cure options	0.008 *	0.000	0.003
Social interactions		0.003 *	0.216 ***
Emotional support		0.160 ***	0.004 **
Informational support		0.005 ***	0.243 ***
Community status			0.210 ***
Social interactions × Community status			−0.916 ***
Emotional support × Community status			−0.020
Informational support × Community status			−0.629 ***
df	3	6	10
F	87.958	214.528	158.784
R^2^	0.066	0.258	0.300
Adjusted R^2^	0.066	0.257	0.299
$\Delta R^{2}$		19.2%	23.4%

Note: * *p* < 0.1, ** *p* < 0.05, *** *p* < 0.001. Unstandardized regression coefficients are reported.

**Table 6:** Results of negative binomial regression.

Dependent Variables	Patient Engagement			
	Baseline Model	Main Effects	Mediation	Moderation
Constant	−1.080 ***	−1.080 ***	−1.093 ***	−1.082 ***
Patient gender	0.003	0.001	0.001	0.000
Registration time	0.025	0.001	0.001	0.000
Cure options	0.024	0.010 *	0.009 *	0.009 *
Social interactions		0.411 ***	0.410 ***	0.565 ***
Emotional support		0.015 **	0.015 **	0.013 **
Informational support		0.589 ***	0.585 ***	0.638 ***
Involvement			0.025 *	
Community status				0.603 **
Social interactions × Community status				−2.409 **
Emotional support × Community status				−0.088
Informational support × Community status				−1.853 **
Log likelihood	−2218.973	−2218.567	−2218.566	−2218.468
Prob > chi2	0.9589	0.000	0.000	0.000
Pseudo R^2^	0.000	0.000	0.000	0.000
# Obs	3709	3709	3709	3709

Note: * *p* < 0.1, ** *p* < 0.05, *** *p* < 0.001. Unstandardized regression coefficients are reported.

**Table 7:** Results of alternative measurement of patient engagement.

Dependent Variables	Patient Engagement (Volume of Patient Reply)			
	Baseline Model	Main Effects	Mediation	Moderation
Constant	0.013 ***	0.013 ***	0.009	0.013 ***
Patient gender	−0.001	−0.001	−0.001	−0.001
Registration time	0.050 *	0.048 *	0.048 *	0.050 *
Cure options	0.005 *	0.001	0.001	0.002
Social interactions		0.019 *	0.019 *	0.020 *
Emotional support		0.006 *	0.006 *	0.006 *
Informational support		0.159 ***	0.158 ***	0.221 ***
Involvement			0.007 *	
Community status				0.162 **
Social interactions × Community status				−0.209 ***
Emotional support × Community status				0.046
Informational support × Community status				−0.433 ***
df	3	6	6	10
F	77.456	42.285	36.268	31.325
R^2^	0.059	0.065	0.065	0.079
Adjusted R^2^	0.059	0.063	0.063	0.076
$\Delta R^{2}$		0.4%	0.4%	1.7%

Note: * *p* < 0.1, ** *p* < 0.05, *** *p* < 0.001. Unstandardized regression coefficients are reported.

**Table 8:** Results of different samples.

Dependent Variables	Patient Engagement			
	Baseline Model	Main Effects	Mediation	Moderation
Constant	0.342 ***	0.346 ***	0.317 ***	0.350 ***
Patient gender	0.001	0.000	0.000	−0.001
Registration time	0.000	0.012	−0.014	−0.020
Cure options	0.012 *	0.004	0.005	0.005 *
Social interactions		0.137 *	0.132 ***	0.225 ***
Emotional support		0.008 **	0.007 *	0.003 *
Informational support		0.196 ***	0.192 ***	0.121 **
Involvement			0.058 ***	
Community status				0.343 **
Social interactions × Community status				−1.696 ***
Emotional support × Community status				0.128
Informational support × Community status				−0.064 **
df	3	6	6	10
F	14.647	51.153	46.149	44.084
R^2^	0.047	0.258	0.268	0.334
Adjusted R^2^	0.044	0.253	0.262	0.326
$\Delta R^{2}$		20.9%	21.8%	28.7%

Note: * *p* < 0.1, ** *p* < 0.05, *** *p* < 0.001. Unstandardized regression coefficients are reported.

## Data Availability

The data is unavailable due to privacy restrictions.
